# Long forgotten perfume bottle nozzle in the uterus: challenges in retrieval in a low resource setting!

**DOI:** 10.1093/jscr/rjaf053

**Published:** 2025-02-19

**Authors:** Manisha Chhetry, Jyotsna Yadav, Aashish Baniya

**Affiliations:** Department of Obstetrics and Gynecology, BPKIHS, Dharan, 56700, Nepal; Department of Obstetrics and Gynecology, BPKIHS, Dharan, 56700, Nepal; BPKIHS, Dharan 56700, Nepal

**Keywords:** foreign body, foul-smelling discharge, uterine cavity, hysteroscopy, laparotomy

## Abstract

Foreign body in the uterus may lead to severe complications and pose significant management dilemmas. We report a 26-year-old lady who presented with foul-smelling discharge, subfertility, and a failed attempt at foreign body removal outside. Pelvic ultrasound revealed an impacted structure in the endocervical canal while a descending pipe was visualized in the endocervical canal per speculum examination. Partial removal vaginally and surgical removal of the nozzle via laparotomy was necessary due to the size, location, and impaction of the object. Posterior uterine incision was used due to easy accessibility. The post-operative stay was uneventful. This case underscores the importance of early detection, imaging, and multidisciplinary management in cases of uterine foreign bodies.

## Introduction

Foreign bodies in the uterine cavity, though rare, pose significant health risks due to potential complications like infection, perforation, abscess formation, and infertility in rare cases [1–5]. Such cases often go unnoticed until symptoms like vaginal discharge, pelvic pain, bleeding, or subfertility manifest. This case presents the unusual retrieval of a retained perfume nozzle from the uterus of a 26-year-old woman, highlighting the diagnostic dilemmas and challenges in management.

## Case presentation

**Figure 1:**
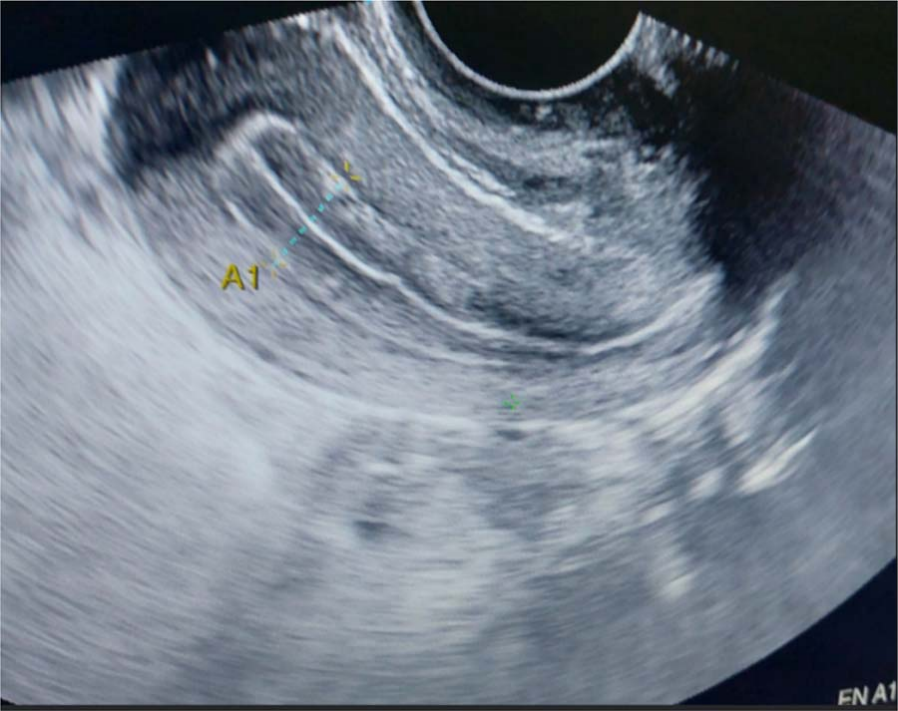
Endometrial thickness grossly normal.

**Figure 2:**
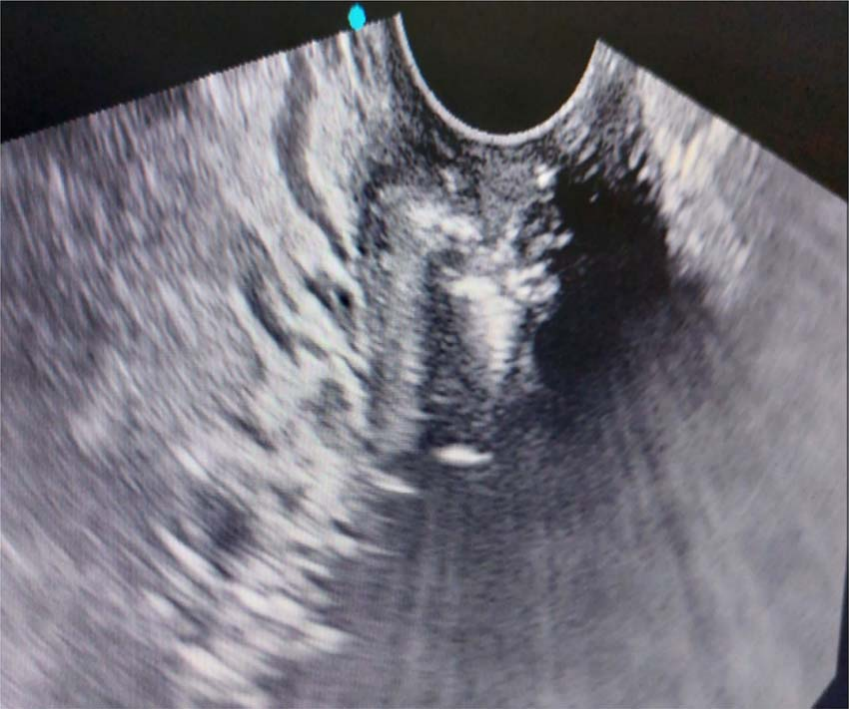
Foreign body seen in the endocervical canal.

**Figure 3:**
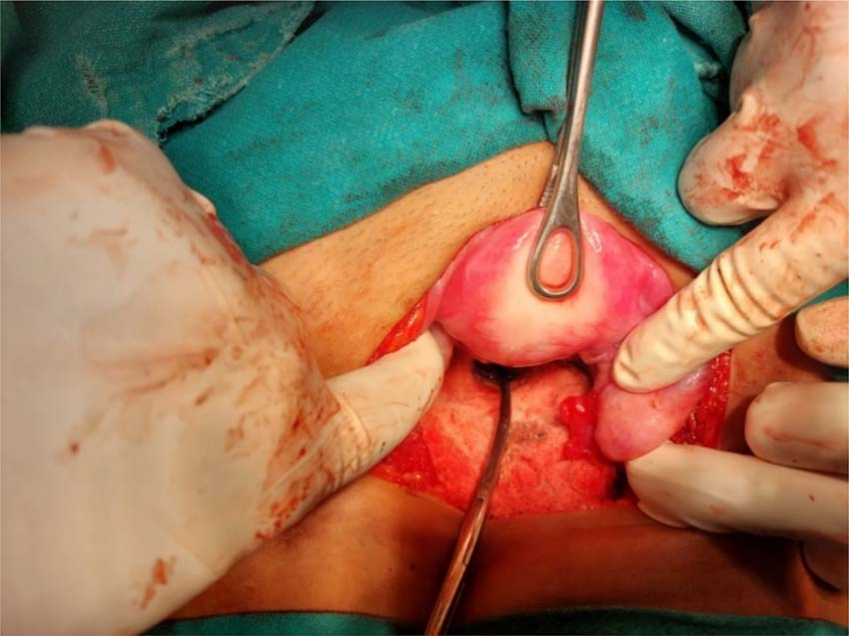
Laparotomy revealing the nozzle head in the lower uterine cavity.

**Figure 4:**
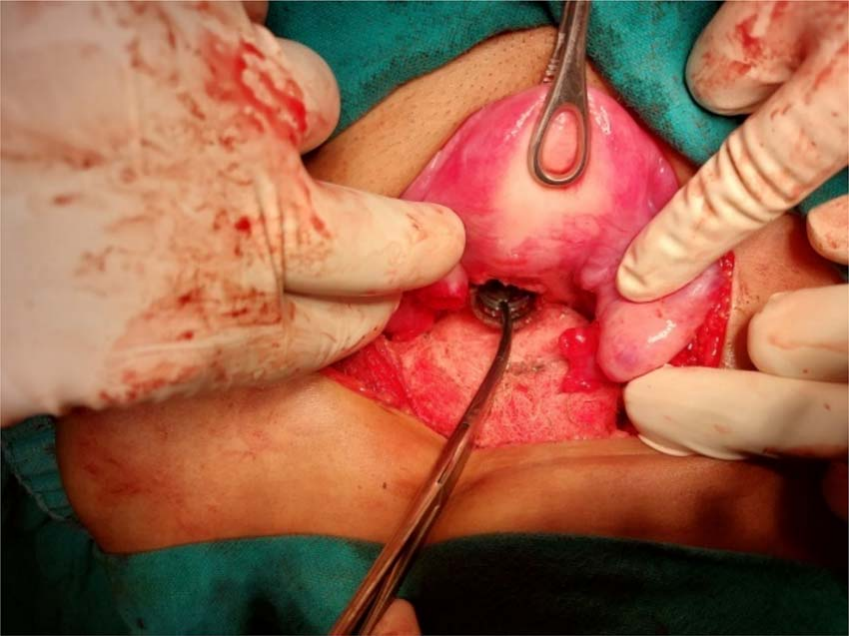
Extraction of the foreign body through a posterior hysterotomy incision.

**Figure 5:**
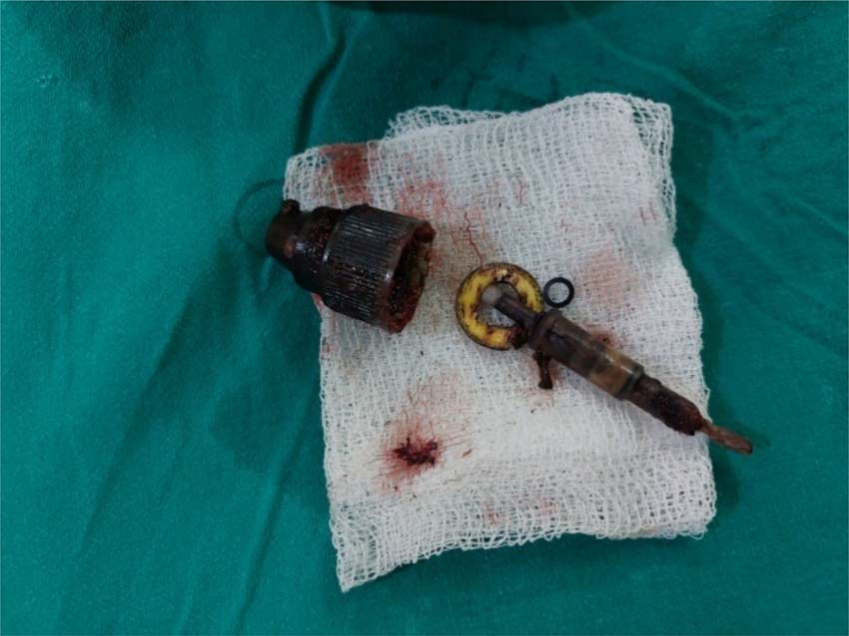
The perfume nozzle head retrieved from posterior hysterotomy and the nozzle with ring and spring removed vaginally.

A 26-year-old nulliparous lady from a rural area was referred to emergency from a primary health care centre with a failed attempt to remove an impacted uterine foreign body. On per speculum examination, an impacted hard pipe-like structure was protruding from the cervix. The patient gave a history of foreign body insertion 9 years back, however, failed to provide a detailed account of the mode of insertion or history of sexual assault. She went to seek medical care only after developing profuse foul-smelling discharge and chronic pelvic pain. Removal was attempted under local anaesthesia but failed. Transvaginal scan showed a bulky uterus with an apparently normal endometrial thickness and echotexture; however, the cervical canal was distended with an echogenic area and areas of calcification (Figs 1 and 2). She was taken up for removal of the foreign body under general anaesthesia. As a hard pipe-like structure was already protruding, removal was tried by grasping it with a Kocher’s forceps. After multiple attempts part of the foreign body was removed. Hysteroscopy showed that the lower endocervical canal was clear and a ring-like structure occupied the upper endocervical canal. Removal under direct vision using a hysteroscopic grasper was attempted but failed due to the impaction and size of the object and the hysteroscope could not be negotiated beyond the object. A laparotomy was performed and after an assessment of anatomy and tactile sensation to assess accessibility, incision on the posterior surface of the uterus was given (Figs 3 and 4) and the object was removed (Fig. 5). The incision was closed in two layers. Intercede was placed at the repair site. An extended course of antibiotics was given for 7 days. Post-operative recovery was uneventful. Contraception for at least 1 year, the need for proper antenatal care, the risk of rupture, and mandatory caesarean delivery were counselled to the couple.

## Discussion

Foreign bodies in the female reproductive tract have been reported across all age groups [5]. The incidence in extremes of age may be higher due to the insertion of objects during play in children however the possibility of sexual abuse should always be kept in mind; meanwhile, in elderly dementia, psychiatric illness could be contributory factors. In young adults, retrieval of foreign bodies like forgotten tampons, IUDs, match sticks, incense sticks, unabsorbed sutures, and retained foetal bones has been reported [2–4, 6].

Forgotten foreign bodies in the uterus have been occasionally, associated with serious complications including pelvic abscess, pyometra, and uterine perforation. The foul-smelling discharge, a classic sign of infection [2] and chronic pelvic pain [7], prompts medical attentionlike in our patient. History and a high index of clinical suspicion help to clinch the diagnosis. Investigations like ultrasonography, magnetic resonance imaging, computed tomography, and X-ray in radio-opaque foreign bodies [8] can assist in locating the foreign body and evaluating the extent of associated complications, such as uterine perforation or abscess formation [3].

Surgical intervention is often required for the removal of deeply embedded foreign objects [3]. In this case, hysteroscopy was used as the initial modality to extract the foreign object, but surgical extraction via laparotomy was necessary due to its size and location. For objects that are small and confined to the uterine cavity like misplaced CuT, hysteroscopic removal is the gold standard [9].

Surgical intervention presents unique challenges and key surgical principles have to be followed to optimize outcome. The need for abdominal procedure either laparoscopy or laparotomy should always be counselled preoperatively; especially if an impacted, large, or migrated foreign body is anticipated where vaginal removal is difficult or unsafe. When considering an incision on the uterus for extraction, the site of the incision should be determined depending on the accessibility of the foreign body. In our case, a posterior transverse incision was chosen as the foreign body was felt pressing the posterior wall. Transverse uterine incisions are easier to repair especially if laparoscopy with ipsilateral port position is opted for. The repair of the uterine incision should comply with principles similar to myomectomy repair: closing dead space, perfect haemostasis, baseball suturing for serosal layer closure, and use of anti-adhesion agents like oxidized regenerated cellulose form mainstay of repair [10, 11]. Postoperatively, use of broad-spectrum antibiotic should be considered and counselling the patient regarding the need for supervised antenatal care, discussion regarding probable problems that could come in future pregnancy like remote risk of rupture uterus should be done.

## Conclusion

This case highlights the importance of thorough clinical evaluation in cases of vaginal discharge, particularly when there is a history of foreign body insertion. The successful surgical removal of the foreign body complying with basic surgical principles highlights the importance of a systematic approach. Foreign bodies retained in the uterine cavity can pose serious health risks, and timely multidisciplinary management is crucial for favourable outcomes.
